# Antioxidant Antagonises Chemotherapeutic Drug Effect in Lung Cancer Cell Line A549

**DOI:** 10.31557/APJCP.2020.21.4.1019

**Published:** 2020-04

**Authors:** Swetha Rajendran, Aishwarya Lakshminarayanan, Gnanasambandan Ramanathan, Suresh Kannan Subramanian Shanmugam

**Affiliations:** *Department of Biomedical Sciences, Sri Ramachandra Institute of Higher Education and Research, Chennai, India. *

**Keywords:** Lung cancer, A549 cell line, antioxidant, chemotherapeutic drug

## Abstract

**Objective:**

This study aimed to find whether antioxidants increase or decrease the effect of chemotherapeutic drug in the* in vitro* model.

**Methods:**

Small lung Cancer cell line (A549) was treated with anticancer drug 6-Thioguanine (6-TG) at different concentration viz., 1, 10, 50 and 100μM and the proliferation was measured using MTT assay. The antioxidant N-Acetyl Cysteine (NAC) in different ratios viz., 1mM, 5mM and 10mM were assayed for their effect in proliferation on the A549 cells alone and in combination with 6-TG.

**Results:**

Our experiment proves that anticancer drug 6-TG decreases the proliferation and the antioxidant NAC enhances the proliferation of A549 cells. Strikingly when co-treated with 6-TG, the antioxidant NAC diminished the proliferation reduction action of 6-TG on A549 cells.

**Conclusion:**

Our results suggest that antioxidants in fact benefit the tumor cell growth when treated alone and when in combination with anticancer drug, it severely impair the activity of the drug. We propose that extreme care should be taken when prescribing antioxidants alone or in combination with chemotherapeutics.

## Introduction

Antioxidants are compounds which has the ability to scavenge the unstable and highly reactive free radicals. Endogenous antioxidants produced by the body due to increased generation of reactive oxygen species neutralise the activity of ROS, thereby preventing the oxidative stress induced tissue damage. As we age, endogenous antioxidant system shows decreased efficiency in scavenging free radicals. Hence, many age associated diseases such as neurodegenerative disorders, cancer, etc are associated with increased ROS production (Pawelec et al., 2014). These highly reactive ROS are potent inducers of damage in biomolecules (Sies, 2007). ROS can cause direct DNA damage such as oxidation and methylation of bases leading to spontaneous mutation and mitochondrial dysfunction that are oncogenic (Leon et al., 2016). It also oxidises amino acids, resulting in improper folding of protein, protein-protein cross linkage and protein fragmentation which ultimately leads to the accumulation of dysfunctional protein and Endoplasmic Reticulum (ER) stress (Niforou et al., 2014). Hence, many approaches using antioxidants are arising every day to decrease or prevent oxidative stress induced cell damage and cell death in healthy and diseased conditions. On special view from cancer therapy, many antioxidants are used as radio- and chemo- sensitizers and protectors (Mehrali, 2015; Thyagarajan and Sahu, 2018). 

Chemotherapy, the preferred method of treating cancer, suffer from its own adverse effects especially due to the build-up of oxidative stress. The concept of antioxidants can help fight cancer is continuously promoted and use of antioxidant supplement during cancer therapy is increasing. Few studies claims that carotenoids and retinoids can suppress the proliferative phenotype of cancer cells, revert premalignant condition and prevent malignant progression (Teicher BA, 1994). The antioxidant Resveratrol potentiates Cisplatin – an anticancer drug* in vivo* and *in vitro *via G2/M phase cell cycle arrest, apoptosis and autophagy pathways and also prevents the nephrotoxicity and cytotoxicity of cisplatin (Lee et al., 2016). Ascorbic acid when administered intravenously in high and frequent doses, inhibits in colorectal cancer with KRAS mutation by inhibiting key enzymes in glycolysis pathway (El Halabi et al., 2018). Several research groups study the interference of antioxidants with the chemotherapeutic drug and the results are contrary in nature. The cytotoxicity of doxorubicin on breast cancer cells (MCF-7) can be reduced or abolished by chemical and enzymatic antioxidants (Doroshow, 1986). Tangeretin, a flavonoid with antioxidant property on combination with tamoxifen, neutralises the growth inhibition of cancer by tamoxifen in breast cancer patients (Bracke et al., 1999). The important big question is whether the antioxidant is good or bad in increasing the efficacy of chemotherapeutic drugs.

N-Acetyl Cysteine (NAC) is a precursor for L-cysteine that in turn contributes to the synthesis of glutathione. It is used in the treatment of chronic obstructive pulmonary disorder (COPD) and polycystic ovarian disease (PCOD) due to its mucolytic activity (Mokhtari et al., 2017). NAC has showed reduction in Notch3 levels through lysosome-dependent protein degradation, thereby negatively regulating Notch3 malignant signalling in Notch3-expressing tumors (Zhang et al., 2016). 

The drug 6-Thioguanine (6-TG) exhibit anticancer and immune-suppressive activities in both* in vivo* and* in vitro* studies. 6-TG is commonly used to treat wide variety of cancers including lung cancer, leukemias, lymphomas,melanoma, glioblastoma and soft tissue sarcoma (Vokes et al., 1992).The anticancer activity is based on the conversion of this drug into purine analogue by the action of hypoxanthine-guanine phosphoribosyl transferase (HGPRT) that get incorporated into DNA leading to DNA damage and replication arrest (Coulthard and Hogarth, 2005).This drug is also well tolerated in the patients with lung cancer as evident by a phase II clinical study on advanced non-small lung cancer patients (Vokes et al., 1992).* In vitro*, 6-TG has been shown to arrest the growth of leukemic cell lines (Morgan et al., 1994).

In this study, we aim to find whether antioxidants increase or decrease the effect of anticancer drug. 

## Materials and Methods

*Culturing of A549 cells*

The A549 cells were purchased from National centre for Cell Sciences (NCCS), Pune, India and cultured in DMEM media constituted with 10% FBS and 100 units/ml of penicillin, 100 mg/ml of streptomycin. The cells were passaged when they reach 80% confluence using Trypsin EDTA enzyme and are used for proliferation assays.

*MTT preparation*

The MTT reagent was prepared fresh every time just before the assay. The MTT was purchased from Himedia and the stock solution of MTT was prepared by adding 5mg of MTT to 10 ml of 1X PBS and stored in aluminium foil wrapped tubes at 4^o^C. For experiments, 100µl from this stock solution was added to each well of 96 well plates for assay.

*NAC preparation*

A stock solution of 100 mM of NAC was prepared by weighing 163mg of NAC (Himedia) and dissolved in 10ml of 1X PBS solution under sterile condition. The solution was filtered using 0.2µm PVDF membrane and stored in 4^o^C. The working stock of 100 μM was prepared for assay using serum free DMEM media. 

*6-TG preparation*

A stock solution of 1.5 mM of 6-TG (50X) was prepared by weighing 2.5mg of 6-TG and dissolved in 10 ml of serum free media (DMEM) under sterile condition. The solution was filtered using 0.2µm PVDF membrane and store at 4^o^C. The working stock of 150 μM was prepared for assay using serum free media.

*MTT Assay*

For MTT assay, the cells were seeded onto the 96 well plates at the density of 7000 cells/well. The cells were incubated overnight in CO2 incubator. The following day, cells in the plate were treated with the different concentrations of antioxidant, anticancer agent and/or combined concentrations of anticancer drug and antioxidant. NAC was tested in 1mM, 5mM and 10mM doses (Sayin et al., 2014) and 6-TG at 1, 10, 50 and 100μM doses (Mender et al., 2015). The cells were maintained in the incubator for 48 hours after the treatment. After the incubation time, the media and drug was completely removed and 100µl of freshly prepared MTT was added to each well. The plate was incubated at room temperature in dark room for 4 hours. After incubation, the wells were observed under the microscope for the formation of insoluble formazon crystals. The crystals were dissolved by adding 100µl of DMSO. After incubation for 1 hour in dark room, the wells were read by microplate reader at 590nm.

*Statistical analysis*

The means from three different experiments were considered for calculation and the level of significance is calculated by t-test. The error bars represented in the graph are standard deviations.

## Results

*Anticancer agent 6-TG decreases the proliferation of Cancer cell line A549*

**Figure 1:**
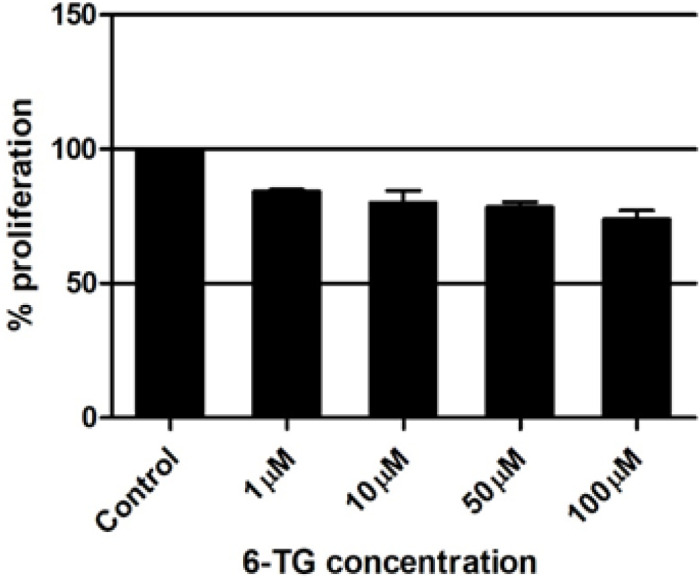
Effect of 6-TG on A549 Cell Lines. 6-TG, 6-Thioguanine

Analysing the MTT data, we found out that the anticancer drug 6-TG decreases the proliferation of lung cancer cells i.e., A549 in a dose depended manner. The 6-TG was tried in four different concentration viz., 1, 10, 50 and 100μM. All the concentrations showed a significant difference in the proliferation reduction ranging from 16% at 1μM and with a maximum reduction of 26% with 100μM dose(p value < 0.02).This dose depended significant reduction was observed in all the triplicate experiments. The mean was used to plot the graph and the error bars represents the standard deviation (Figure 1).

*The antioxidant NAC increases the proliferation of Cancer cell line A549*

**Figure 2:**
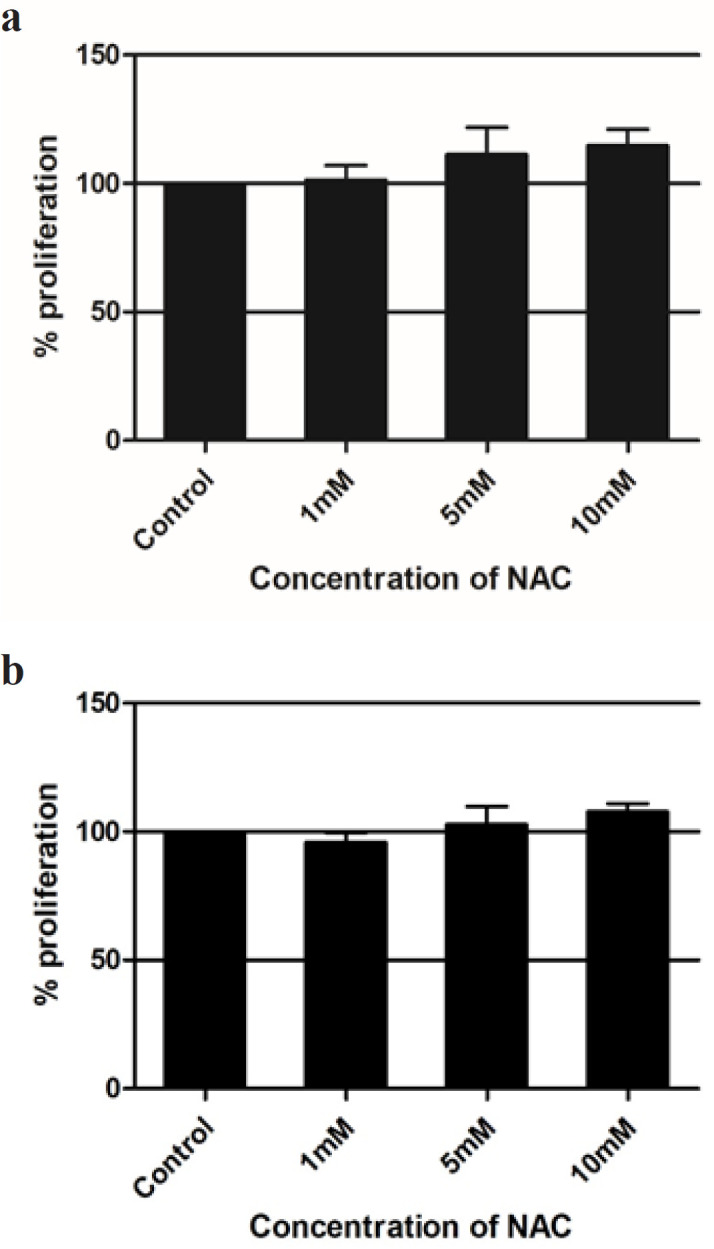
a, Effect of NAC on A549 Cell Lines; b, Effect of NAC on CHO cells. NAC, N-Acetyl Cysteine; CHO, Chinese Hamster Ovary

To find out the action of antioxidant on the cancer cells, the cells were treated with different concentrations of N-Acetyl Cysteine (NAC). Cells without any treatment serve as the control. The test wells were treated with 1mM, 5mM and 10mM of NAC. We noticed that the antioxidant increases the proliferation of cancer cells in a dose dependent manner.Though there is no significant changes, there is a marked increase in the proliferation of A549 cells compared to untreated. A maximum of 15% increase in the proliferation was observed with 10mM of N-Acetyl Cysteine treatment (Figure 2a). This experiment was repeated in triplicates and the mean was used to plot the graph. To compare the activity of NAC on the non-cancer cell line, CHO cell lines were treated with similar concentrations of NAC and we observe a dose depended increase in proliferation of these cells too (Figure 2b).

*Effect of 6-TG is negatively regulated by antioxidant treatment*

**Figure 3:**
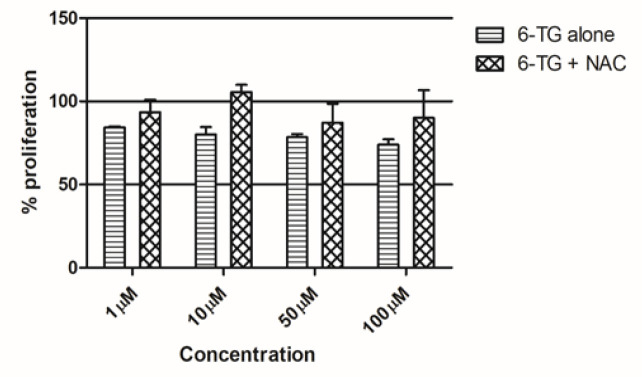
Combinational Effect of 6-TG and NAC. 6-TG, 6-Thioguanine; NAC, N-Acetyl Cysteine

As a next step, we checked the combined action of antioxidants and cancer drug on the A549 cells. The cells were treated with the combinations of both 6-TG and NAC. Cells without any treatments act as the control. The concentration of NAC that gave the maximum effect was fixed and used with different concentrations of 6-TG. The results indicate an overall decrease in proliferation of A549 cells treated with anti-oxidant and 6-TG, but when compared to the 6-TG alone, there was an increase in proliferation observed at all doses.When co-treated the growth retarding effect of 6-TG at all the concentrations was altered in a negative way by NAC (Figure 3). The difference was also significant at the dose of 10μM of 6-TG (p = 0.029).

## Discussion

Antioxidants have an important role as free radical scavengers in the human body. Antioxidants are commonly taken as food supplement by healthy people and also prescribed as part of medication in case of various diseases especially for cancer patients. In our study we check the effect of antioxidant (NAC) and anticancer drug (6-TG) alone and in combination on the* in vitro* proliferation of small lung carcinoma cell line (A549). 

The drug 6-TG is widely used as a preferred drug of choice with broad spectrum of activity to treat variety of cancers like leukemias, lymphomas, melanoma, glioblastoma etc. including lung cancer (Vokes et al., 1992; Munshi et al., 2014). Our results with 6-Thioguanine (6-TG) is as expected have negative effect on the proliferation of A549 cells on the dose dependent manner.A wide range ofconcentration was used ranging from 1μM to 100 μM and we observed a significant decrease in proliferation of the A549 cells in all the concentrations.

When we compared the effect of antioxidants (NAC) alone and in combination with anticancer drug (6-TG), we observed interesting results. The antioxidant NAC increases the proliferation of A549 cells in a dose depended manner. Though the results is not significant, NAC increases the proliferation of A549 cells in all the tested doses viz 1mM, 5mM and 10mM.When cotreated with 6-TG, the growth reducing effect of 6-TG was inversely effected by NAC. At all the treated concentrations, NAC brings down the effect to 6-TG and there was a significant change at 10 μM concentration of 6-TG.

There are mixed results been reported on the effect of antioxidants on the reduction of cancer (Patterson et al., 1997). Different types of antioxidants show contradictory results in various types of cancers. A negative correlation has been reported in a case-control study between antioxidants and several cancers. Also several randomised controlled clinical trials showed no relation between dietary antioxidants and primary cancer prevention including lung cancer (Alpha-Tocopherol, 1994; Omenn et al., 1996). A systematic review analysing the different databases and clinical trials between 2005 and 2013 reveal no benefit from antioxidants supplements on prevention of cancer (Fortmann et al., 2013). An* in vivo* study in mice reveal that the antioxidant, NAC and Vitamin E in fact promote the progression of lung cancer and reduce their survival by disturbing the P53 pathway (Sayin et al., 2014).

In Conclusion, our results also indicates that antioxidant (NAC) increases the proliferation of small lung cancer cell line – A549 and when combined with the anticancer drug 6-TG, it downregulate the activity of 6-TG by the evidence of increased proliferation above to the drug treated cells. Overall, our results suggest a negative effect of antioxidants alone or in combination with anticancer drug in reducing the proliferation of lung cancer cell line A549. Further studies are required to assess the effect of this combination in other types of cancers and to elucidate their mode of action. It is of prior importance and with careful attention that the antioxidant supplementation to cancer therapeutics to be looked at.

## References

[B1] Alpha-Tocopherol BCCPSG (1994). The effect of vitamin E and beta carotene on the incidence of lung cancer and other cancers in male smokers. N Engl J Med.

[B2] Bracke ME, Depypere HT, Boterberg T (1999). Influence of tangeretin on tamoxifen’s therapeutic benefit in mammary cancer. J Natl Cancer Inst.

[B3] Coulthard S, Hogarth L (2005). The thiopurines: an update. Invest New Drugs.

[B4] Doroshow JH (1986). Prevention of doxorubicin-induced killing of MCF-7 human breast cancer cells by oxygen radical scavengers and iron chelating agents. Biochem Biophys Res Commun.

[B5] El Halabi I, Bejjany R, Nasr R (2018). Ascorbic acid in colon cancer: From the basic to the clinical applications. Int J Mol Sci.

[B6] Fortmann SP, Burda BU, Senger CA (2013). Vitamin and mineral supplements in the primary prevention of cardiovascular disease and cancer: An updated systematic evidence review for the US Preventive Services Task Force. Ann Intern Med.

[B7] Lee YJ, Lee GJ, Yi SS (2016). Cisplatin and resveratrol induce apoptosis and autophagy following oxidative stress in malignant mesothelioma cells. Food Chem Toxicol.

[B8] Leon J, Sakumi K, Castillo E (2016). 8-Oxoguanine accumulation in mitochondrial DNA causes mitochondrial dysfunction and impairs neuritogenesis in cultured adult mouse cortical neurons under oxidative conditions. Sci Rep.

[B9] Mehrali JPIaH (2015). Lycopene as a carotenoid provides radioprotectant and antioxidant effects by quenching radiation-induced free radical singlet oxygen: An overview. Cell J.

[B10] Mender I, Gryaznov S, Dikmen ZG (2015). Induction of telomere dysfunction mediated by the telomerase substrate precursor 6-thio-2’-deoxyguanosine. Cancer Discov.

[B11] Mokhtari V, Afsharian P, Shahhoseini M (2017). A review on various uses of N-Acetyl Cysteine. Cell J.

[B12] Morgan CJ, Chawdry RN, Smith AR (1994). 6-Thioguanine-induced growth arrest in 6-mercaptopurine-resistant human leukemia cells. Cancer Res.

[B13] Munshi PN, Lubin M, Bertino JR (2014). 6-thioguanine: a drug with unrealized potential for cancer therapy. Oncologist.

[B14] Niforou K, Cheimonidou C, Trougakos IP (2014). Molecular chaperones and proteostasis regulation during redox imbalance. Redox Biol.

[B15] Omenn GS, Goodman GE, Thornquist MD (1996). Effects of a combination of beta carotene and vitamin A on lung cancer and cardiovascular disease. N Engl J Med.

[B16] Patterson RE, White E, Kristal AR (1997). Vitamin supplements and cancer risk: the epidemiologic evidence. Cancer Causes Control.

[B17] Pawelec G, Goldeck D, Derhovanessian E (2014). Inflammation, ageing and chronic disease. Curr Opin Immunol.

[B18] Sayin VI, Ibrahim MX, Larsson E (2014). Antioxidants accelerate lung cancer progression in mice. Sci Transl Med.

[B19] Sies H (2007). Biological redox systems and oxidative stress. Cell Mol Life Sci.

[B20] Teicher BA SJ, Holden SA, Ara G, Northey D (1994). In vivo modulation of several anticancer agents by beta-carotene. Cancer Chemother Pharmacol.

[B21] Thyagarajan A, Sahu RP (2018). Potential contributions of antioxidants to cancer therapy: Immunomodulation and radiosensitization. Integr Cancer Ther.

[B22] Vokes EE, Lyss AP, Herndon JE (1992). Intravenous 6-thioguanine or cisplatin, fluorouracil and leucovorin for advanced non-small cell lung cancer: a randomized phase II study of the cancer and leukemia group B. Ann Oncol.

[B23] Zhang X, Wang YN, Zhu JJ (2016). N-acetylcysteine negatively regulates Notch3 and its malignant signaling. Oncotarget.

